# Supplementary material for: Common Running Musculoskeletal Injuries and Associated Factors among Recreational Gorge Marathon Runners: An Investigation from 2013 to 2018 Taroko Gorge Marathons
Source: Int J Environ Res Public Health. 2020 Nov 3;17(21):8101. doi: 10.3390/ijerph17218101 (PMC7663022; doi:10.3390/ijerph17218101)
Supplement: Supplementary file 1 [file ijerph-17-08101-s001.pdf]

International Journal of
Environmental Research and Public Health

MDPI

Supplementary file: Questionnaires

Sports Injury Survey Questionnaire

1. Do you have experience in participating in the marathon: □No; □Yes, □<1 year □1-2 years □ 2-3 years □3-4 years □4-5 years □More than 5 years (marathon/half marathon also please note)

2. Number of times to participate in marathon: average_____times/per week; ____per month; ____per year.

3. Normal running times: approximately___times/week or month; each time approximately____ kilometers or minutes.

4. Do you have experience in running a mountain road marathon: □No. □Yes, about times.

   a. Have you received special training or planned training? □No; □Yes,

   b. Training method:____
   Frequency:____times/week; about____kilometers or ______ hours each time.

5. Have you ever had sports injuries or pain (sore) problems:

   □No; □Yes, in □Ankle joint □Knee joint □Hip joint □Low back

   □Shoulder and neck joint□Upper limbs (elbows, wrists) □feet or toes □Calf muscles (tendons)□

   Thigh muscles (tendons)□Back muscles □Shoulder and neck muscles □Others: (multiple selection possible)

6. Is there any pain (sore) after this run:

   □No;□ Yes, in □Ankle joint □Knee joint □Hip joint □Low back □Shoulder and neck joint □

   Upper limbs (elbows, wrists) □feet or toes □Calf muscles (tendons) □Thigh muscles

   (tendons)□Back muscles □Shoulder and neck muscles □Others:_____(multiple choice)

7. Before this run, you spend about minutes warming up.

   warm up method: □Massage□stretch□Tapping□Other______(multiple choice),

*site: □ankle joint □knee joint □hip joint □lower back □shoulder and neck joint □upper limb (hand, Elbows, wrists) □Feet or toes Calf muscles /tendons □Thigh muscles/tendons/) □ Lower back muscles□Shoulder and Neck muscles □Others:______(multiple choice)

8. In your usual experience of running a marathon (including half marathons), the Taroko (Mountain Road) Marathon and the Flatland Marathon will affect you: □No effect, □ Influence:

a. Location: □ankle joint □knee joint □hip joint □lower back □shoulder and neck joint □upper limb (elbow, wrist) □feet or toe □calf muscle (tendon) □thigh muscle (tendon) □lower back muscle □shoulder and neck muscles □other: (multiple choice)

b. □ It takes more time to warm up than usual, about ________minutes.

9. Do you know that the conference provides sports injury physical therapy services: □Yes; □No

10. You feel about the sports injury physiotherapy services provided by the conference: □not satisfied □not satisfied □good (normal) □somewhat satisfied □very satisfied.

20** Taroko Gorge Marathon
Sports injury and physical therapy record sheet

Participant Number:_______ Gender: Male Female Age:________ Height:________ Weight:_______ Participating Mileage: Half Full

Education level: ☐ Elementary ☐Junior High ☐ High School ☐Junior College or Above ☐Graduate School

Sports injury history: ☐None ☐Yes, site (indicated in the table below), first occurrence to date: ______years/______month/_______week,

experienced process: continuous intermittent,

Have you received diagnosis and treatment: No Yes. Diagnosis: ___________________________ Treatment: _______________________________

Sports injury Hx.site ,pain site and treatment site	protection and treatment items
**black**: Sports injury Hx.**red**: Pain **blue**: Treatment site<br>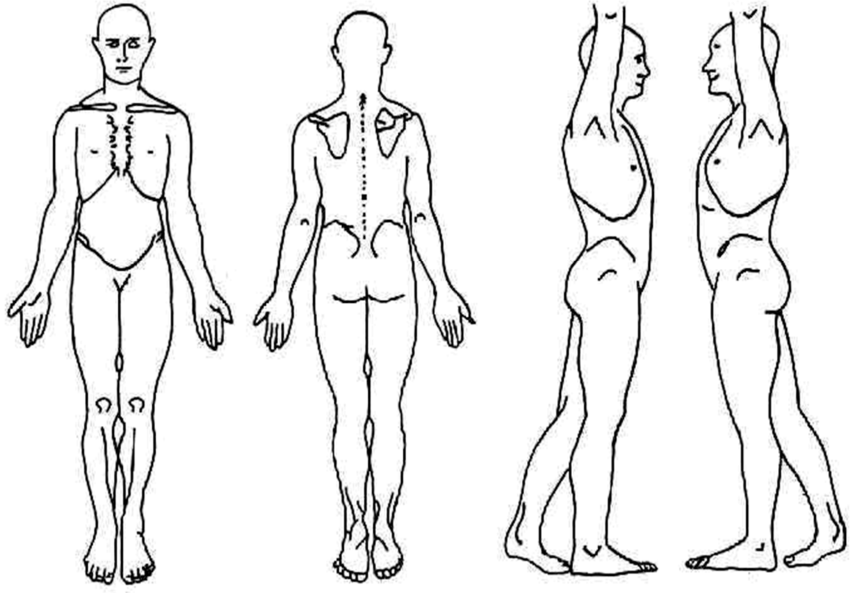	Ice pack: ☐Ice bag x____ ☐Ointment ☐Cold spray x_____<br>Massage: ☐soft tissue massage and DFM ☐nerve point massage☐lymphatic massage<br>Stretch: ☐manual stretch ☐education stretch<br>Site: ☐Quadriceps ☐Posterior thigh muscles☐ITB ☐Adductors femoris☐Gluteal muscles ☐Iliopsoas ☐Piriformis☐Back muscles ☐Anterior Calf muscles☐Posterior calf muscles☐Anterior neck muscles ☐Posterior neck muscles☐ Pectoralis muscles☐Rotator cuff

	Joint mobilization: ☐Lumbar and sacral spine☐Hip joint☐Knee joint<br>☐Ankle joint☐cervical-thoracic spine☐Shoulder joint ☐Elbow joint<br>☐Wrist joint<br>Tapping: ☐Athletic tape ☐Bandage ☐Leuko tap ☐Kinesio tap<br>Site: ☐feet ☐ankle ☐calf ☐knee ☐thigh ☐other_____<br>other: ☐ running shoe testing ☐ exercise guidance and pain treatment consultation ☐_____________<br>VAS scale: (pre-) /10, (post-) /10<br>GROC: -7, -6, -5, -4, -3, -2, -1, 0, +1, +2, +3, +4, +5, +6, +7<br>(-7: A very great deal worse，0: About the same，+7: A very great deal better)

Signature of name:________________________
